# Concomitant Mutations G12D and G13D on the Exon 2 of the KRAS Gene: Two Cases of Women with Colon Adenocarcinoma

**DOI:** 10.3390/diagnostics11040659

**Published:** 2021-04-06

**Authors:** Elena De Falco, Luca Pacini, Daniela Bastianelli, Gian Paolo Spinelli, Chiara Spoto, Enzo Veltri, Antonella Calogero

**Affiliations:** 1 Department of Medical Surgical Sciences and Biotechnologies, Sapienza University of Rome, 04100 Latina, Italy; antonella.calogero@uniroma1.it; 2 Mediterranea Cardiocentro, 80122 Napoli, Italy; 3 Clinical Pathology Unit, ICOT Hospital, Sapienza University of Rome, 04100 Latina, Italy; l.pacini@giomi.com (L.P.); d.bastianelli@giomi.com (D.B.); 4 UOC Territorial Oncology, AUSL Latina–CdS Aprilia, Sapienza University of Rome, 04011 Aprilia, Italy; gianpaolo.spinelli@uniroma1.it; 5 Medical Oncology, Santa Maria Goretti Hospital, 04100 Latina, Italy; c.spoto@ausl.latina.it (C.S.); e.veltri@ausl.latina.it (E.V.)

**Keywords:** colorectal cancer, next generation sequencing, KRAS, G12D, G13D

## Abstract

Colorectal cancer (CRC) is rapidly increasing representing the second most frequent cause of cancer-related deaths. From a clinical-molecular standpoint the therapeutically management of CRC focuses on main alterations found in the RAS family protein, where single mutations of KRAS are considered both the hallmark and the target of this tumor. Double and concomitant alterations of KRAS are still far to be interpreted as molecular characteristics which could potentially address different and more personalized treatments for patients. Here, we firstly describe the case of two patients at different stages (pT2N0M0 and pT4cN1cM1) but similarly showing a double concurrent mutations G12D and G13D in the exon 2 of the KRAS gene, normally mutually exclusive. We also evaluated genetic testing of dihydropyrimidine dehydrogenase (DPYD) and microsatellite instability (MSI) by real-time PCR and additional molecular mutations by next generation sequencing (NGS) which resulted coherently to the progression of the disease. Accordingly, we reinterpreted and discuss the clinical history of both cases treated as single mutations of KRAS but similarly progressing towards a metastatic asset. We concluded that double mutations of KRAS cannot be interpreted as univocal genomic alterations and that they could severely impact the clinical outcome in CRC, requiring a tighter monitoring of patients throughout the time.

## 1. Introduction

Sporadic colorectal cancer (CRC) represents one of the most increasing type of tumor of the last decades, as the result of significant changes in our lifestyle habits—including dietary factors, smoking, obesity, and sedentary behavior—acknowledged to modulate epigenetics [1]. However, the 5–6% of CRC is associated to susceptibility to polyposis, playing a key role in both surveillance and diagnosis of CRC in this set of subjects [2]. Despite this, epidemiological worldwide studies are continuing to strengthen the concept that differences among populations and even between men and women in terms of survival rate, are likely ascribable to genetic background factors. In fact, from a molecular point of view, the genomic asset of patients with CRC, is crucial to identify the best treatment and to tailor a personalized targeted therapy. 

Colorectal cancer can be the result of accumulated changes in chromosome alterations even in absence of family history, confirming that the genome is a unique hallmark in patients and so it should be interpreted. 

KRAS, a small GTPase belonging to the RAS family including also NRAS and HRAS, is considered the most mutated gene in all cancer types and the “gold” biomarker in CRC [3]. This is expected, as the RAS proteins control several biological processes from cell proliferation and differentiation to migration and survival, by alternating in on and off states, a process finely regulated by guanine nucleotide exchange factors (GEFs) and GTPase activating proteins (GAPs) [4,5]. Consequently, deregulations of the RAS family play a crucial role in cancer progression towards metastatic phases [4,6,7]. Moreover, KRAS also represents the predictive factor for the successful employment of monoclonal antibody against EGFR such as cetuximab and panitumumab [8,9].

Although RAS proteins display high homology within the family but the C-terminus, mutations of KRAS, NRAS, and HRAS are currently considered not equivalent. Additional studies are demonstrating that even in the same gene of the RAS family including KRAS, mutations cannot be conceived as similar as they differently affect the clinical outcome of the disease and response to chemotherapy [10,11]. Accordingly, we acknowledge that in CRC, KRAS mutations of codons 12, 13, and 61 preferentially occur at high frequency, but additional mutations of codons such as 19, 117, and 146 may exist at low frequency.

Although the abovementioned oncogenic changes are the result of missense mutations (85%) [12] with gain of function effect on the protein (they generate a constitutive GTPase activity with the loss of any control by the GAPs [3]), they do differ in terms of specific biochemical pathways and downstream effectors involved [13,14]. This suggests that we should reinterpret the oncogenic changes of KRAS (and of the whole RAS family) in the light of the clinical history of the patient and discriminate these tiny differences that seem apparently irrelevant. Moreover, the significance of double and/or concomitantly oncogenic mutations of KRAS in patients with CRC remains unclear.

Although double mutations are rare events, the biological and clinical implication are not yet understood. Here, we report two women affected by CRC showing two concurrent mutations G12D and G13D on the exon 2 of the KRAS gene, which are normally mutually exclusive in patients. Given that patients harbor identical KRAS mutations, we have reinterpreted the double genetic asset with patients’ clinicopathological features and clinical history.

## 2. Results

Between March 2019 and November 2020, a total of 132 cases with CRC were referred to our laboratory for in depth analysis and to investigate molecular genetic mutations for potential targeted therapy. Out of 132 cases screened by next generation sequencing, two female patients (Patient A and Patient B, 78 and 58 years old, respectively) displayed the double mutation reported in this study. Patient A underwent surgery for obstructing carcinoma of the cecum. The histopathological analysis showed an ulcerative and moderately differentiated adenocarcinoma staged as pT2N0M0 with mucinous aspects (<5%). The neoplasm infiltrated the muscular tunic where neoplastic lymphangitis was present. According to the stage, a standard follow-up was started until metacron hepatic, pulmonary, and lymph nodes metastasis were found by computed tomography (CT) scan. Patient B was admitted for a massive metastatic disease showed by CT with lymph-nodes and more than 20 liver metastatic lesions, mainly in the right hepatic lobe. For this reason, a palliative surgery and biopsies of colon vegetative lesion was performed. The histopathological examination displayed an ulcerative adenocarcinoma staged as pT4cN1cM1.

Afterwards, biopsies from both patients were evaluated by mutational analysis. Paraffin-embedded tissue sections were collected on microscopic slides and stained for hematoxylin and eosin in order to select the tumor area by optical microscopy. After manual dissection of the cancer area, genomic DNA was extracted using the Gene-Read DNA FFPE KIT (Qiagen, Milan, Italy) according to the manufacturer’s instruction. DNA concentration (12.6 and (12.6 and 26 ng/μL for patient A and B, respectively) was determined by Qubit4 fluorometer with Quant-IT dsDNA HS Assay Kit (Invitrogen). Library construction with patient’s DNA including quality and quantity was performed using 40 ng of DNA by employing GeneRead QIAact AIT DNA UMI Panel [15], QIAxcell DNA high resolution kit (Qiagen) and Qubit4 fluorometer with Quant-IT dsDNA HS Assay Kit (Invitrogen), respectively. Sequencing was performed by GeneReader platform (Qiagen) according to the manufacture’s protocols. Primary sequence data were first processed by QCI Analyze for GeneReader1.5.0 software (Qiagen) and clinical SNVs were detected by Ingenuity software (Qiagen). The application was internally designed and developed by QIAGEN. All analyses were based on: QIAGEN Clinical Insight-Interpret (7.1.20201218), Ingenuity Knowledge Base (B-release), CADD (v1.6), Allele Frequency Community (2019-09-25), EVS (ESP6500SI-V2), Refseq Gene Model (2020-04-06), JASPAR (2013-11), Ingenuity Knowledge Base Snapshot Timestamp (29 January 2021), Vista Enhancer hg18 (July 2012), Vista Enhancer hg19 (July 2012), Clinical Trials (B-release), MITOMAP: A human Mitochondrial Genome Database http://www.mitomap.org.2019 (Accessed on 2020-06-19), PolyPhen-2 (v2.2.2), 1000 Genome Frequency (phase3v5b), ExAC (0.3.1), iva (Nov 20 02:39), TargetScan (7.2), PhyloP hg18 (NCBI36 (hg18) 2019-11, GRCh37 (hg19) 2014-02, GRCh38 2015-05), PhyloP hg19 (NCBI36 (hg18) 2019-11, GRCh37 (hg19) 2014-02, GRCh38 2015-05),

GENCODE (Release 33), CentoMD (5.3), OMIM (26 July 2020), gnomAD (2.1.1), BSIFT (23 February 2016), TCGA (5 September 2013), Clinvar (15 September 2020), DGV (15 May 2016), COSMIC (v92), HGMD (2020.4), OncoTree (oncotree_2019_03_01), dbSNP (NCBI36 (hg18) 151, GRCh37 (hg19) 153, GRCh38 153), SIFT4G (2016-02-23). Weekly updates to Ingenuity Knowledge Base for clinical trials recruitment status and new findings from recent articles. Variants are reported according to HGVS nomenclature and were classified following ACMG guidelines. The genes analyzed (the whole CDS was covered as well as the intronic sequences (10-bp) immediately flanking the exons) for SNVs were: *AKT1, ALK1, BRAF, CTNNB1, DDR2, EGFR, ERBB2, ERBB3, ERBB4, ESR1, FBXW7, FGFR1, FGFR2, FGFR3, FLT3, GNA11, GNAQ, HRAS, KIT, KRAS, MAP2K1, MAP2K2, MET, NOTCH1, NRAS, PDGFRA, PIK3CA, RAF1, SMAD4, SOD2,* and *STK11.* The genes analyzed for CNV was: *ALK, BRAF, EGFR, ERBB2, FGFR1, FGFR2, FLT3, KIT, KRAS, MAP2K1, MET,* and *PI3KCA.* For the read coverages and the Average Quality scores of UMI reads for each patient, see Supplementary material (Tables S1, S2 and Supplementary Figure S1).

**Figure 1:**
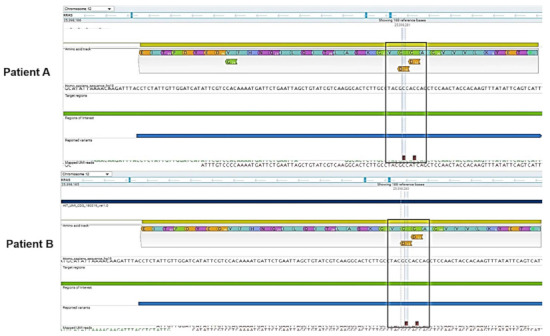
Next generation sequencing analysis of Patient A and B displaying in the exon 2 of the KRAS gene the double nucleotide changes c.38G>A (amino acid change p.G13D) and c.35G>A (amino acid change p.G12D) of pathogenic significance. The black box highlights both mutations.

Results (Figure 1) have shown that both Patients exhibited two coexistent mutations on the same exon 2 of the KRAS gene with the nucleotide changes c.38G>A (amino acid change p.G13D) and c.35G>A (amino acid change p.G12D) both classified with pathogenic significance. The effect of the parallel changes at two different amino acid positions resulted as gain of function mutation. The variant allelic fraction (VAF) was estimated 34% (p.G12D) and 31% (p.G13D) for Patient A and 4.6% (p.G13D) and 12.6% (p.G12D) for Patient B. More intriguingly, the NGS analysis also showed that numerous additional mutations were present. Both patients exhibited changes in the PIK3CA gene. Patient A showed the pathogenic significant nucleotide change c.317G>T (amino acid change p.G106V) on the exon 2 of the PIK3CA gene with a VAF of 12% and gain of function effect on the corresponding protein, whereas Patient B displayed the variant c.1173A>G (amino acid change p.I391M) on the exon 7 of the gene with a VAF of 52.23% and classified as of uncertain significance.

Specifically, Patient A exhibited a molecular profile for SMAD4 similarly to KRAS and of pathogenic significance. Accordingly, exon 9 and exon 12 of the SMAD4 gene were found mutated with the nucleotide change c.1067C>T (amino acid change p.P356L) and c.1610A>G (amino acid change p.D537G), respectively. However, differently from the of the KRAS mutation on the proteins, the two changes on the SMAD4 gene were loss of function mutations. The VAF for the first mutation on SMAD4 was 18% and the second 33%.

Additional variants of uncertain significance were shown by both patients but on different genes. Specifically, Patient A exhibited the genetic variant c. 4598A>G (amino acid change p.D1533G) with a VAF of 45% on exon 26 of the NOTCH1 gene as well on the exon 17 of the ERBB2 gene with a nucleotide change c.1963A>G (amino acid change p.I655V) and VAF of 58%. Patient B showed expressed the nucleotide change c.1562G>A (amino acid change p.R521K) on the exon 13 of the EGFR with a VAF of 18.8% and classified as of uncertain significance.

The NGS confirmed that both Patients were wild type for BRAF, HRAS, and NRAS and for the remaining genes of the molecular profile panel analyzed. Besides, Patient B showed a variant of uncertain significance of the EGFR, differently from Patient A with wild type EGFR. A summary of the whole molecular profile for both subjects is displayed below in Table 1.

Moreover, to investigate more deeply the genetic role of this double mutations, both women were also screened for germline microsatellite instability (MSI), given that MSI is ascribable to 15–20% of patients with CRC [16] as well as in familiar and hereditary forms [17] (during anamnesis Patient A referred of cancer familiarity). Accordingly, we analyzed the full panel of genes including BAT-25, BAT-26, NR-21, NR-24, and NR-27 (recommended by ESMO [18]) and a further group NR-22, CAT-25, and MONO-27. Both patients showed MSI stability with a wild-type genotype. According to the overall molecular tests, patients were similarly treated and prior administration of drugs containing 5-Fluorouracil (FU), the genetic dihydropyrimidine dehydrogenase (DPYD) variants such as c.1236G>A (rs56038477), c.1679T>G (rs55886062), c.1905+1G>A (rs3918290), c.2194G>A (rs1801160), and c.2846A>T (rs67376798) [19] were evaluated. Patients exhibited a wild-type genotype for all genes investigated. Patient A received a first-line Bevacizumab plus Folfox chemotherapy. Partial response was the best overall response. Due to the persistence of stable disease, after 12 cycles of induction chemotherapy, Patient A started a maintenance treatment with Bevacizumab plus De Gramont regimen. To date, maintenance treatment is still ongoing with substantially stable disease. Nevertheless, the last CT scan has already highlighted the progression of the tumor in the hepatic district, although PET analysis will be required as confirmation. Patient B received the same Bevacizumab plus Folfox regimen both as first-line and maintenance treatment.

## 3. Discussion

In CRC three main molecular mechanisms including alteration of the mismatch repair (MSI), aberrant DNA methylation and genetic mutations of main genes, play a key role in neoplastic transformation and progression. According to different guidelines, the mutational analysis of the RAS family is not recommended at early stages (I and II), where the MSI analysis is rather preferred, as several studies indicate a good correlation to discriminate for a possible adjuvant therapy [20,21].

KRAS is mainly considered a predictive factor for the employment of anti-EGFR monoclonal antibodies in combination with chemotherapy. Thus, although not confirmed in all studies, the role of KRAS as prognostic factor is mainly conceivable for metastatic stages, highlighting the need of deeper investigations on this topic [9,22,23]. Notably, mutations of KRAS account for the 40% of patients with CRC [24] and they have represented the intense effort of drug targeting over the last decades. Very recently, the AMG510 inhibitor for the KRAS^G12C^ (glycine to cysteine) mutation is currently under clinical trial phase I for lung adenocarcinoma, demonstrating that KRAS is a druggable target gene [25,26]. Similarly, in 2018 the FDA has approved the use of MRTX849, a novel and covalent inhibitor for the specific KRAS^G12C^ mutation [27,28], highlighting the role of KRAS as a distinct and independent driver mutation.

Our two cases underline the significance of the double mutations of KRAS. To date, there are no specific diagnostic algorithms to further stratify patients based on the presence of double mutations. Thus, from a clinical standpoint, a patient harboring a single mutation of KRAS is similarly treated as a patient with concurrent genomic alterations. Although double mutations on the same exon of KRAS are very rare events, our report suggests that the double mutations could be considered as a distinct subset of genomic alterations and that they should be investigated even at early stages to attempt the prediction of a potential progression of the cancer.

According to the clinical guidelines recommending the combination of chemotherapy with biological agents as first-line treatment, both patients were treated with the same therapeutical regimen, normally employed for the metastatic stages (a combination of fluoropyrimidines, oxaliplatin, and bevacizumab). In fact, the use of biological agents has been demonstrated to increase the standard chemotherapy efficacy as Chen et al. showed the role of the addition of bevacizumab to chemotherapy regimens (XELOX, FOLFIRI, Folfox), highlighting a significant improvement of progression free survival (PFS; HR = 0.68; 95% CI = 0.59–0.78; *p* < 0.00001) [29].

Thus, we could suggest that the screening of double mutations of KRAS would be useful not only to detect the potential resistance to anti-EGFR monoclonal antibody, but more importantly to identify patients at high-risk as Patient B. Hence, this set of subjects will benefit from an intensified follow up similarly to patients with established metastatic stages (as Patient A). Other recent studies have also suggested that the inclusion of different cohorts of patients with multiple mutations would be useful to identify potential pharmacological targets and to better understand the physiopathological meaning of these genomic alterations [30].

Importantly, in colorectal cancer cells the G12D and G13 D KRAS mutation have been recently demonstrated to activate distinct and independent signaling pathways, resulting in dissimilar biological effects [31]. Similarly, different cohorts of patients with lung adenocarcinoma, have highlighted that KRAS mutations are extremely heterogenous and that co-alterations are present at high frequency [32]. A further study has corroborated these observations for additional emerging predictive and prognostic biomarkers. Specifically, coexisting mutations of KRAS and SMAD4 or TP53 worsen both the overall survival and relapse free survival compared to the single mutation of KRAS [33]; therefore, authors concluded that KRAS alone cannot be considered the only predictive prognosis after resection of colorectal liver metastases.

Coherently with a metastatic profile, we also observed in Patient A but not Patient B concomitant mutations of SMAD4 (loss function) and PIK3CA with pathogenic significance. PIK3CA and SMAD 4 have been involved in drug resistance to anti-EGFR monoclonal antibodies [34] and to 5-fluoruracil mediated apoptosis in cell lines [35], respectively. SMAD4 loss mutations has been found to positively associated to mucinous CRC (Patient A had a mucinous CRC) [36], and progression of CRC, because of its key role to control the TGFβ1-SMAD4 axis of the epithelial-mesenchymal transition [37]. These late-stage modifications have been found in Patient B in line with the metastatic profile. Notably, we could not unequivocally assess in this study the role of SMAD-4 and PIK3CA mutations beyond KRAS. The prognostic significance of both markers as driver mutations in CRC in absence of KRAS mutations has not yet fully established.

Although conclusive data to validate multiple mutations are not available to adopt different clinical strategies, and so far, our patients have not shown an enhanced resistance to the same therapeutical regimen, however the concomitant presence of SMAD4 and PIK3CA could be strengthen a potential future resistance effect due to the G12D and G13D KRAS mutations, especially in Patient B.

Besides, the parallel investigation of the MSI analysis has been also important to add prognostic information in combination with the molecular profile. MSI and chromosome instability are two main mechanisms in the etiopathogenesis of CRC. Notably, both mechanisms can coexist or being mutually exclusive [38]. Recently, it has been reported a correlation between MSI status, KRAS mutation and clinical-pathological features of patients with CRC at stage III [39], showing the strict association between high MSI and high mutation rate of KRAS (with the mutation rate of p.G12D in codon 12 of exon 2 the highest, followed by p.G13D). Conversely, low MSI or MSS are associated with low mutation rate of KRAS. Besides, MSI-high has been reported as the mostly frequent in female patients with stage II and III [40] and in subjects aged ≥ 50 years. Moreover, observational studies have described the association between MSI and KRAS mutation as the hallmark of a worse prognosis of mucinous CRC with metastatic profile [41,42]. Thus, wild type or mutated KRAS always in association with microsatellite stability, is a negative predictor for disease survival [43,44]. Besides, the presence of hereditary settings in MSI can be linked to KRAS mutations or to the Lynch syndrome. As both patients were female, aged ≥ 50, with a mucinous/ulcerative stage II and IV CRC, MSI was performed. Although, a high MSI was expected, both patients were stable.

The MSI analysis was also determined in order to provide the best pharmacological treatment for both patients. In fact, we reckon that the variable prognostic value of KRAS mutations in operable CRC [45,46], should be accompanied by the MSI analysis, as this latter better correlates with the adjuvant therapy in patients with stage II and III [47].

Notably, MSI has been also associated with high-rate response to immunotherapy after employment of PD-1/PDL-1 inhibitors in CRC [48,49,50], therefore strengthening the MSI investigation as a novel important parallel predictive marker. In our two cases, the double mutation in KRAS was not accompanied by alterations of microsatellites in the germline, therefore potentially suggesting the propensity to develop sporadic CRC.

In conclusion, the analysis of the double mutations in the RAS family should be evaluated not as univocal genomic alterations, but rather as accumulation of biological events which should be carefully monitored throughout the time.

## Figures and Tables

**Table 1:** Molecular variant details of patients analyzed by next generation sequencing (NGS)

Patient	Gene	Exon	Nucleotide Change	Amino Acid Change	Allele Fraction (%)	Classification	Effect on Protein
A	*KRAS*	2	NM_004985.5: c.38G>A	p.G13D	31	Pathogenic	Gain of function
A	*KRAS*	2	NM_004985.5: c.35G>A	p.G12D	34	Pathogenic	Gain of function
A	*SMAD4*	9	NM_005359.5: c.1067C>T	p.P356L	18	Pathogenic	Loss of function
A	*SMAD4*	12	NM_005359.5: c.1610A>G	p.D537G	33	Pathogenic	Loss of function
A	*PIK3CA*	2	NM_006218.3: c.317G>T	p.G106V	12	Pathogenic	Gain of function
A	*ERBB2*	17	NM_004448.3: c.1963A>G	p.I655V	58	VUS	Gain of function
A	*NOTCH1*	26	NM_017617.5: c.4598A>G	p.D1533G	45	VUS	Loss of function
B	*KRAS*	2	NM_004985.5: c.38G>A	p.G13D	4.6	Pathogenic	Gain of function
B	*KRAS*	2	NM_004985.5: c.35G>A	p.G12D	12.6	Pathogenic	Gain of function
B	*EGFR*	13	NM_005228.5: c.1562G>A	p.R521K	18.8	VUS	Loss of function
B	*PIK3CA*	7	NM_006218.4: c.1173A>G	p.I391M	52.23	VUS	Normal function

VUS: variant of uncertain significance

## Supplementary Materials

The following are available online at https://www.mdpi.com/article/10.3390/diagnostics11040659/s1. Table S1: Reads coverage patient A, Table S2: Reads coverage patient B, Figure S1: Average Quality scores of UMI reads.
