# Supplementary material for: Concomitant Mutations G12D and G13D on the Exon 2 of the KRAS Gene: Two Cases of Women with Colon Adenocarcinoma
Source: Diagnostics (Basel). 2021 Apr 6;11(4):659. doi: 10.3390/diagnostics11040659 (PMC8067479; doi:10.3390/diagnostics11040659)

Patient A

Table S1. Reads coverage

Name	ROI	Bases	≥100x	≥60x	0x	Median	VOI	VOI <100x	VOI <60x
NRAS	3	456	100.00%	100.00%	0.00%	5,510	51	0	0
DDR2	7	1,335	100.00%	100.00%	0.00%	7,087	0	0	0
ALK	17	2,650	100.00%	100.00%	0.00%	4,714	52	0	0
ERBB4	8	1,142	100.00%	100.00%	0.00%	7,231	0	0	0
RAF1	2	8	100.00%	100.00%	0.00%	4,938	2	0	0
CTNNB1	1	230	100.00%	100.00%	0.00%	9,094	0	0	0
PIK3CA	18	1,130	100.00%	100.00%	0.00%	6,141	165	0	0
FGFR3	5	800	100.00%	100.00%	0.00%	2,970	274	0	0
PDGFRA	12	452	100.00%	100.00%	0.00%	7,037	52	0	0
KIT	13	1,047	100.00%	100.00%	0.00%	7,378	280	0	0
FBXW7	5	878	100.00%	100.00%	0.00%	6,215	0	0	0
TERT	0	0					0		
ESR1	5	24	100.00%	100.00%	0.00%	7,753	11	0	0
SOD2	0	0					0		
EGFR	21	1,362	100.00%	100.00%	0.00%	5,220	599	0	0
MET	9	2,512	100.00%	100.00%	0.00%	8,697	0	0	0
BRAF	4	270	100.00%	100.00%	0.00%	6,018	526	0	0
FGFR1	6	838	100.00%	100.00%	0.00%	4,938	0	0	0
GNAQ	2	263	100.00%	100.00%	0.00%	8,599	2	0	0
NOTCH1	2	585	100.00%	100.00%	0.00%	2,267	0	0	0
FGFR2	4	458	100.00%	100.00%	0.00%	5,673	204	0	0
HRAS	3	456	100.00%	100.00%	0.00%	4,743	0	0	0
GAPDH	0	0					0		
KRAS	5	464	100.00%	100.00%	0.00%	7,511	162	0	0
ERBB3	8	32	100.00%	100.00%	0.00%	7,464	10	0	0
FLT3	1	3	100.00%	100.00%	0.00%	5,320	1	0	0
Name	ROI	Bases	≥100x	≥60x	0x	Median	VOI	VOI <100x	VOI <60x
AKT1	1	131	100.00%	100.00%	0.00%	6,564	0	0	0
MAP2K1	11	1,204	100.00%	100.00%	0.00%	5,001	3	0	0
ERBB2	14	1,433	100.00%	100.00%	0.00%	4,788	73	0	0
SMAD4	8	1,279	100.00%	100.00%	0.00%	5,788	0	0	0
STK11	4	747	100.00%	100.00%	0.00%	3,034	0	0	0
GNA11	2	263	100.00%	100.00%	0.00%	4,091	2	0	0
MAP2K2	1	213	100.00%	100.00%	0.00%	4,858	0	0	0

Name: Gene name.

ROI: Number of Regions of Interest, i.e. reportable regions that overlap with the gene.

Bases: Total number of base positions in Regions of Interest that overlap with the gene.

≥100x: Percentage of base positions in Regions of Interest that overlap with the gene for which coverage is equal to or above the significant coverage threshold.

≥60x: Percentage of base positions in Regions of Interest that overlap with the gene for which coverage is equal to or above the minimum coverage threshold.

0x: Percentage of base positions in Regions of Interest that overlap with the gene for which coverage is zero.

Median: Median coverage of base positions in the Regions of Interest that overlap with the gene.

VOI: Total number of Variants of Interest, whether detected or not, that overlap with the gene. The list of Variants of Interest is defined by the analysis pipeline.

VOI <100x: Number of Variants of Interest in the gene for which coverage is below the significant coverage threshold.

VOI <60x: Number of Variants of Interest in the gene for which coverage is below the minimum coverage threshold.

Patient B

Table S2. Reads coverage

Name	ROI	Bases	≥100x	≥60x	0x	Median	VOI	VOI <100x	VOI <60x
NRAS	3	456	100.00%	100.00%	0.00%	1,628	51	0	0
DDR2	7	1,335	100.00%	100.00%	0.00%	2,201	0	0	0
ALK	17	2,650	100.00%	100.00%	0.00%	1,602	52	0	0
ERBB4	8	1,142	100.00%	100.00%	0.00%	2,534	0	0	0
RAF1	2	8	100.00%	100.00%	0.00%	1,056	2	0	0
CTNNB1	1	230	100.00%	100.00%	0.00%	3,005	0	0	0
PIK3CA	18	1,130	100.00%	100.00%	0.00%	1,733	165	0	0
FGFR3	5	800	100.00%	100.00%	0.00%	1,071	274	0	0
PDGFRA	12	452	100.00%	100.00%	0.00%	2,169	52	0	0
KIT	13	1,047	100.00%	100.00%	0.00%	2,028	280	0	0
FBXW7	5	878	100.00%	100.00%	0.00%	2,169	0	0	0
TERT	0	0					0		
ESR1	5	24	100.00%	100.00%	0.00%	2,392	11	0	0
SOD2	0	0					0		
EGFR	21	1,362	100.00%	100.00%	0.00%	2,057	599	0	0
MET	9	2,512	100.00%	100.00%	0.00%	2,960	0	0	0
BRAF	4	270	100.00%	100.00%	0.00%	2,136	526	0	0
FGFR1	6	838	100.00%	100.00%	0.00%	1,677	0	0	0
GNAQ	2	263	100.00%	100.00%	0.00%	1,924	2	0	0
NOTCH1	2	585	100.00%	100.00%	0.00%	947	0	0	0
FGFR2	4	458	100.00%	100.00%	0.00%	1,340	204	0	0
HRAS	3	456	100.00%	100.00%	0.00%	1,939	0	0	0
GAPDH	0	0					0		
KRAS	5	464	100.00%	100.00%	0.00%	2,329	162	0	0
ERBB3	8	32	100.00%	100.00%	0.00%	1,397	10	0	0
FLT3	1	3	100.00%	100.00%	0.00%	1,655	1	0	0
Name	ROI	Bases	≥100x	≥60x	0x	Median	VOI	VOI <100x	VOI <60x
AKT1	1	131	100.00%	100.00%	0.00%	1,717	0	0	0
MAP2K1	11	1,204	100.00%	100.00%	0.00%	1,600	3	0	0
ERBB2	14	1,433	100.00%	100.00%	0.00%	1,328	73	0	0
SMAD4	8	1,279	100.00%	100.00%	0.00%	1,911	0	0	0
STK11	4	747	100.00%	100.00%	0.00%	1,270	0	0	0
GNA11	2	263	100.00%	100.00%	0.00%	1,188	2	0	0
MAP2K2	1	213	100.00%	100.00%	0.00%	1,090	0	0	0

Name: Gene name.

ROI: Number of Regions of Interest, i.e. reportable regions that overlap with the gene.

Bases: Total number of base positions in Regions of Interest that overlap with the gene.

≥100x: Percentage of base positions in Regions of Interest that overlap with the gene for which coverage is equal to or above the significant coverage threshold.

≥60x: Percentage of base positions in Regions of Interest that overlap with the gene for which coverage is equal to or above the minimum coverage threshold.

0x: Percentage of base positions in Regions of Interest that overlap with the gene for which coverage is zero.

Median: Median coverage of base positions in the Regions of Interest that overlap with the gene.

VOI: Total number of Variants of Interest, whether detected or not, that overlap with the gene. The list of Variants of Interest is defined by the analysis pipeline.

VOI <100x: Number of Variants of Interest in the gene for which coverage is below the significant coverage threshold.

VOI <60x: Number of Variants of Interest in the gene for which coverage is below the minimum coverage threshold.

Supplementary Figure S1. Average Quality scores of UMI reads

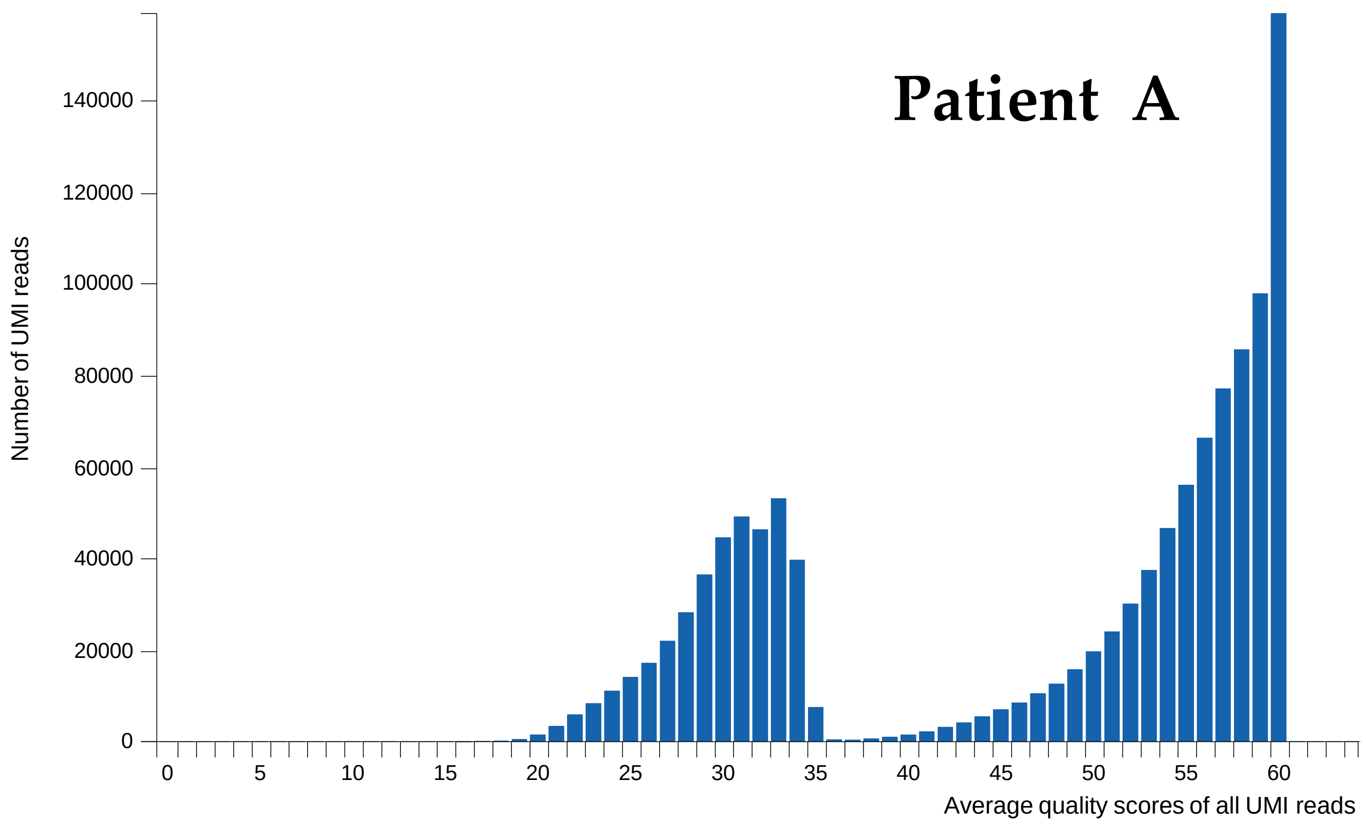

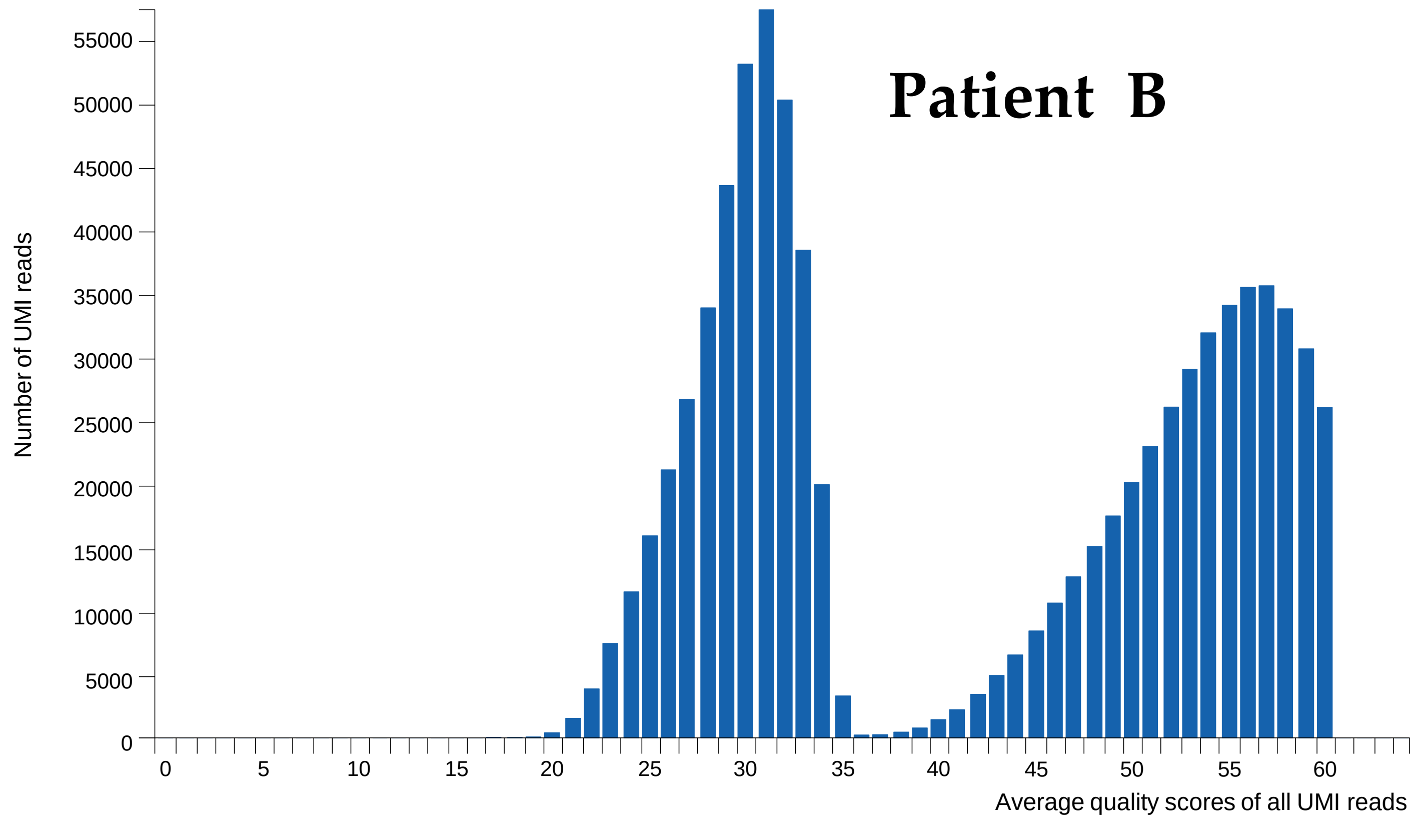

Supplement: Supplementary file 1 [file diagnostics-11-00659-s001.pdf]
